# Critical Care Challenges and Mortality Predictors in Older Adults: A Comprehensive Cohort Analysis

**DOI:** 10.7759/cureus.76433

**Published:** 2024-12-26

**Authors:** João Frutuoso, Francisco Das Neves Coelho, Inês Antunes, Pedro Póvoa

**Affiliations:** 1 Critical Care, Unidade Local de Saúde de Lisboa Ocidental, Lisbon, PRT; 2 Management, Natura Clinica Medica, Lisbon, PRT; 3 Helicopter Emergency Medical Service, Instituto Nacional de Emergencia Medica, Lisbon, PRT

**Keywords:** elderly patients, general critical care, mortality, risk predictors, risk score

## Abstract

Purpose: As the population ages, critically ill older adults increasingly face complications and require more healthcare resources during hospitalization. Since post-ICU (intensive care unit) mortality is an important consideration, particularly in elderly populations, this study aims to assess whether advanced age impacts ICU and post-ICU mortality by comparing outcomes between patients aged 81 years and above with those below 81 years.

Methods: This retrospective study analyzed data from 3,821 ICU patients treated at the Unidade Local de Saúde de Lisboa Ocidental between 2015 and 2023. Key variables included age, gender, ICU length of stay, and severity scores (APACHE [Acute Physiology and Chronic Health Evaluation] II, SAPS [Simplified Acute Physiology Score] II/III, SOFA [Sequential Organ Failure Assessment]). Patients with incomplete records, readmissions, ICU stays shorter than 24 hours, or those under 18 years of age were excluded.

Results: Mortality was significantly higher in patients aged 81 years and above compared to those under 81. Among patients aged 81 and above, ICU mortality was 22% (152 deaths), compared to 13% (342 deaths) in the younger group. Similarly, post-ICU mortality was 20% (138 deaths) for the older group, substantially higher than the 5% (131 deaths) observed in patients below 81 years. The SAPS II and SOFA scores were critical predictors of mortality. Even after adjusting for these scores, older patients still showed higher mortality rates.

Conclusion: This study demonstrated that advanced age is a major factor influencing mortality in critically ill patients, particularly among those aged 81 years and above. These patients faced higher mortality rates both during ICU stays and after discharge, emphasizing the importance of age-specific strategies in managing critically ill elderly populations.

## Introduction

Clinicians are frequently limited in their treatment options by patient’s comorbidities and functional status [1]. Critically ill older adults also tend to consume a higher number of resources and are expected to suffer a higher number of complications and loss of autonomy over the course of their hospitalization [2]. As our population ages, understanding how these elderly patients fare in intensive care units (ICUs) becomes increasingly important. Indeed, older patients may survive and recover well despite critical illness, as previous studies showed [3].

However, common complications from critical illness, such as critical illness polyneuropathy, may lead to long-term disability in elderly patients, as well as longer hospital stays [4].

Functional status after ICU discharge is a key indicator of how well a patient will perform. The better the patient’s performance status is, the lower are the chances the patient may suffer complications or die [5]. However, patient comorbidities and acquired disabilities may significantly affect the outcome. Nearly 20% of patients who initially survive ICU die from complications after discharge within the first six months [6,7]. These late deaths are often linked to limitations on care and decisions made in advance about end-of-life treatment [8].

High-quality discharge processes, including patient education, clear communication among healthcare professionals, and effective organizational tools, are essential [9]. Post-ICU deaths could be prevented through multidisciplinary interventions addressing factors such as off-hours discharge, inadequate rehabilitation, and insufficient sepsis management [10,11]. Most ICU deaths are often attributed to infections, with increased risk among elderly patients, those with pre-existing conditions, and the severity of acute illness at ICU admission, further exacerbated by ongoing organ dysfunction, pre-existing conditions, and the severity of acute illness at ICU admission [12,13].

We considered the question of whether there is a correlation between advanced age and mortality in the ICU and post-ICU discharge. Therefore, we aim to compare mortality rates and risk factors between patients aged 81 years and above versus those below 81 years.

## Materials and methods

Study design and population

We performed a single-center, retrospective study that analyzed data from medical records at the Department of Critical Care Medicine of the Unidade Local de Saúde Lisboa Ocidental from January 2015 to June 2023, where we analyzed all medical processes from adult patients (18 years or older).

Ethical considerations

The study was approved by the relevant ethics committees and institutional review boards in compliance with local regulations. The approval was granted because the research used the data collected for routine clinical practice and posed minimal risk to patients, waiving the requirement for informed consent (2024-55).

Data collection process

Data collected from medical records included gender, age, length of ICU stay, survival status at ICU and hospital discharge, overall hospital stay duration, and APACHE (Acute Physiology and Chronic Health Evaluation) II, SAPS (Simplified Acute Physiology Score) II, SAPS III, and daily SOFA (Sequential Organ Failure Assessment) scores used to assess illness severity. Data collection also covered discharge times and units to provide a comprehensive view of patient trajectories. Follow-up time was considered until the patient was discharged from the hospital.

To ensure the integrity of the included data, we excluded from our sample records with missing at least one critical data point (mortality and SAPS II, SAPS III, and APACHE scores), readmissions within the same episode to avoid duplication, and ICU admissions lasting less than 24 hours to focus on substantial ICU stays.

The dataset provided a robust foundation for analyzing outcomes in elderly ICU patients. Since the scope of this study was to evaluate the mortality outcomes of the very elderly, in and out of ICU until hospital discharge, we divided our patient population into two groups based on age criteria for direct comparison of mortality: patients aged from 18 to 80 years and patients aged 81 and above.

Statistical analysis

Researchers calculated descriptive statistics to summarize the information, including averages and standard deviations for continuous and categorical variables. For categorical variables, they reported frequencies and percentages.

To compare the mortality rates and severity scores between the two age groups (below 81 and 81+), the Mann-Whitney U test was used. Logistic regression models were employed to identify factors influencing death both in the ICU and after discharge. These models considered various severity scoring systems (SAPS II, SAPS III, SOFA at admission and SOFA at discharge, and APACHE II). The researchers compared the performance of these models using Akaike Information Criterion (AIC) values and pseudo-R-squared statistics.

Researchers also adjusted mortality rates to account for illness severity. Specifically, SOFA-adjusted mortality rates were compared between the age groups using regression models and the Wilcoxon rank-sum test. The study also utilized charts tracking mortality rates over time (cumulative mortality curves) to identify trends, including any significant increases or periods of stability across different age groups within the ICU setting. All statistical analyses were performed using RStudio 2024.09.0+375® (Posit PBC, Boston, MA) and SPSS 25® (IBM Corp, Armonk, NY).

## Results

During the study period, 3,821 patients met the inclusion criteria. The average age of the patients was 66 years, with 42% (1,603 patients) being female and 58% (2,218 patients) being male. Among these, 692 patients were aged 81 years or older, with an average age of 85 years, while 2,623 patients were younger than 81 years, with an average age of 61 years (Table 1).

**Table 1:** Population This table presents combined statistical data on demographics, ICU outcomes, and severity scores, along with the frequency distribution of gender among the study population, including subsets for patients aged below 81 years and those aged 81 years and above. ICU, intensive care unit; SOFA, Sequential Organ Failure Assessment; APACHE, Acute Physiology and Chronic Health Evaluation; SAPS, Simplified Acute Physiology Score.

Variable	Overall (n = 3,821)	Age ≥81 (n = 692)	Age <81 (n = 2,623)
Gender			
Female	1,603 patients (42%)	287 patients (41.5%)	1,097 patients (41.8%)
Male	2,218 patients (58%)	405 patients (58.5%)	1,526 patients (58.2%)
Missing	506 patients	-	-
ICU Length of Stay (days)			
Average	9.01 days (95% CI: [8.50, 9.50])	8.06 days (95% CI: [7.20, 8.90])	9.27 days (95% CI: [8.60, 9.90])
Minimum	2 days	2 days	2 days
Maximum	177 days	115 days	177 days
Age (years)			
Average	66 years (95% CI: [65, 67])	85 years (95% CI: [84, 86])	61 years (95% CI: [60, 62])
Minimum	18 years	81 years	18 years
Maximum	104 years	104 years	80 years
Death in ICU	494 patients (15%)	152 patients (22%)	342 patients (13%)
Death after ICU discharge	169 patients (8%)	138 patients (20%)	131 patients (5%)
SOFA Score			
First SOFA	6.54 points (95% CI: [6.00, 7.00])	7.11 points (95% CI: [6.40, 7.80])	6.39 points (95% CI: [5.80, 7.00])
Last SOFA	4.44 points (95% CI: [4.00, 4.90])	5.16 points (95% CI: [4.50, 5.80])	4.25 points (95% CI: [3.70, 4.80])
SAPS II Score			
Average	45.78 points (95% CI: [44.00, 47.50])	55.21 points (95% CI: [52.00, 58.50])	43.29 points (95% CI: [41.00, 45.50])
Minimum	16 points	19 points	0 points
Maximum	118 points	118 points	110 points
SAPS III Score			
Average	44.83 points (95% CI: [43.00, 46.50])	54.24 points (95% CI: [51.00, 57.50])	42.35 points (95% CI: [40.00, 44.50])
Minimum	0 points	34 points	0 points
Maximum	98 points	98 points	98 points
APACHE II Score			
Average	21.04 points (95% CI: [20.00, 22.00])	24.53 points (95% CI: [23.00, 26.00])	20.12 points (95% CI: [19.00, 21.50])
Minimum	0 points	6 points	0 points
Maximum	55 points	51 points	55 points

**Figure 1:**
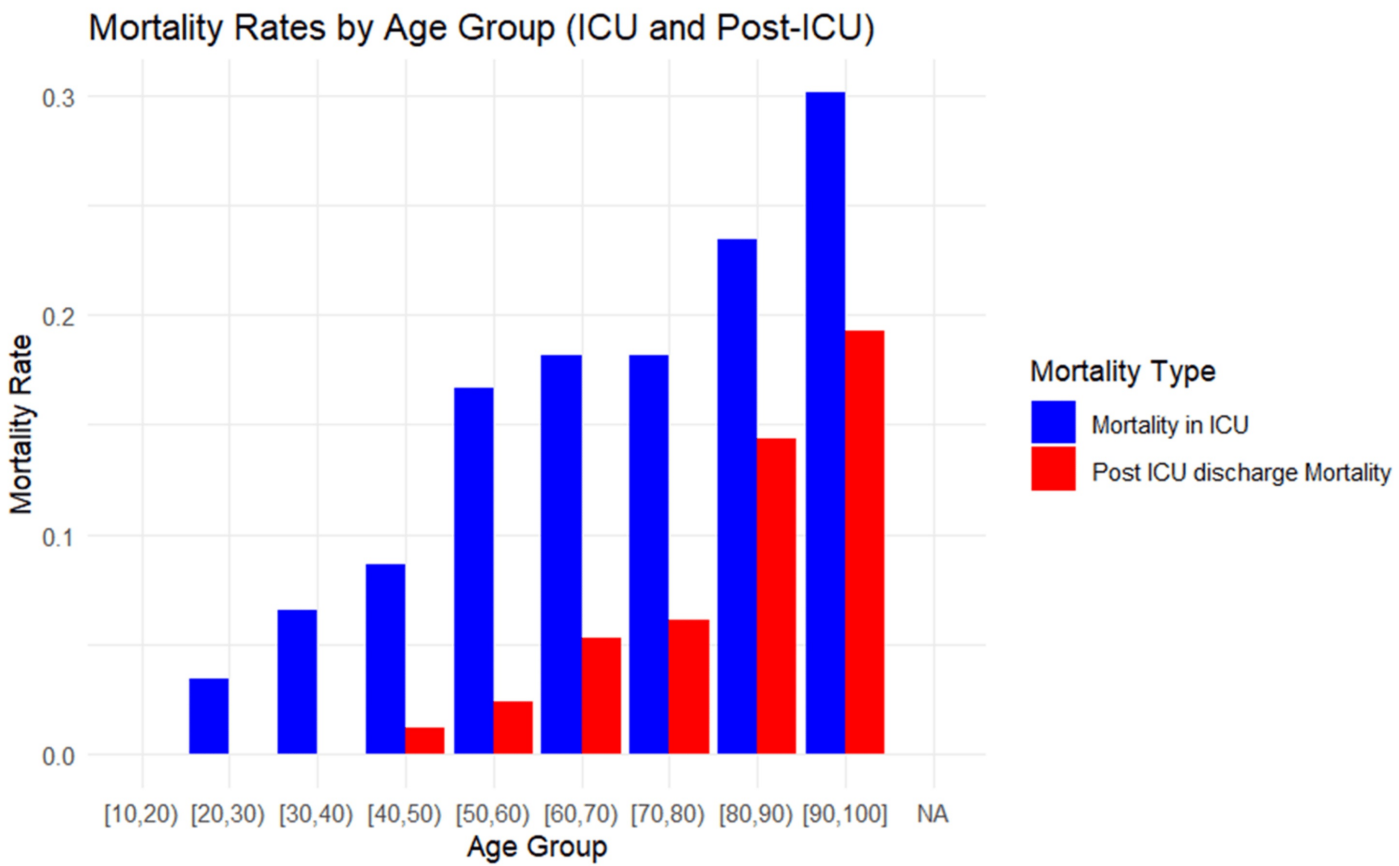
Mortality rates The graph shows a clear trend of increasing mortality rates with advancing age. The lowest mortality rate is observed in the [10,20) age group, and the highest mortality rate is seen in the [90,100] age group. There is a noticeable rise in the mortality rates starting from the [20,30) age group, continuing to increase steadily across the older age groups, reaching a peak in the oldest age category. This graph highlights the significant correlation between higher age groups and increased ICU mortality rates. ICU, intensive care unit.

The lowest mortality rate was observed in the youngest age group, with rates remaining relatively low until the [50,60) age group. From there, mortality rates begin to rise steadily, with a significant increase in the [80,90) age group and the highest rate observed in the oldest age group, suggesting a strong correlation between advancing age and mortality (Figure 1).

**Figure 2:**
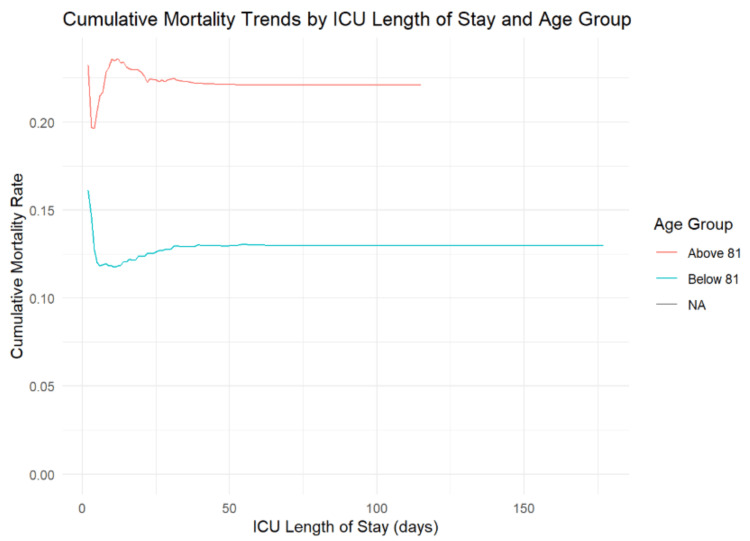
Cumulative mortality This graph highlights that individuals above 81 years have a higher cumulative mortality rate compared to those below 81 years, regardless of the length of stay in the ICU. ICU, intensive care unit.

In ICU settings, individuals above 81 years have a higher cumulative mortality rate, stabilizing around 20% shortly after ICU admission, while those below 81 years stabilize at a lower rate, around 15% (Figure 2). Over time, the cumulative mortality for those above 81 years has shown a sharp rise until 2016, followed by a more gradual increase and a plateau from 2020 to 2022, whereas the <81 years group exhibits a steadier, slower rise. These trends highlight the greater mortality risk faced by older individuals, both during ICU stays and over the years.

In our study, we paid particular attention to a subgroup of 692 patients aged 81 to 104 years. Mortality rates were closely examined both in the ICU and after discharge. In this elderly population, the mortality rates were 22% in the ICU and 20% post-ICU, showing similar outcomes during both periods. Among these patients, 58.5% (405) were male and 41.4% (287) were female.

We investigated mortality rates outside the ICU for patients aged below 81 years and those aged 81 years and above. Using various scoring systems and logistic regression models, we identified significant predictors of mortality, including age and pre-discharge SOFA score. The mortality rate for patients aged 81 or above was significantly higher when compared to patients with 80 years or younger (5% vs 20%, p < 0.001).

When doing the comparative analysis, we found statistically significant differences in both ICU and post-ICU mortality, as well as in severity scores (SOFA, SAPS II, SAPS III, and APACHE II) between patients aged above and below 81 years. Severity and mortality scores were also higher in the older group, with statistical relevance (Table 2).

**Table 2:** Comparative analysis The logistic regression analysis demonstrated that all severity scores significantly predicted both in-ICU and post-discharge mortality. Among these, the SAPS II score emerged as the strongest predictor for in-ICU mortality, with the lowest AIC value of 2475.4, suggesting that it offers the most accurate fit for mortality prediction during the ICU stay. For post-discharge mortality, the SOFA before discharge had the strongest association with outcomes, with a high coefficient estimate of 0.3343 and an AIC of 1408.1, indicating its critical role in predicting long-term survival after ICU discharge. ICU, intensive care unit; SOFA, Sequential Organ Failure Assessment; AIC, Akaike Information Criterion; APACHE, Acute Physiology and Chronic Health Evaluation; SAPS, Simplified Acute Physiology Score.

Score	In-ICU Mortality			Post-Discharge Mortality		
	Coefficient (95% CI)	p-Value	AIC	Coefficient (95% CI)	p-Value	AIC
APACHE II	0.0864 [0.0754, 0.0974]	<0.001	2530.417	0.0471 [0.0328, 0.0614]	<0.001	1570.678
SAPS II	0.0474 [0.0417, 0.0531]	<0.001	2475.422	0.0291 [0.0218, 0.0364]	<0.001	1549.070
SAPS III	0.0556 [0.0482, 0.0630]	<0.001	2561.215	0.0379 [0.0281, 0.0477]	<0.001	1554.296
SOFA Initial	0.193 [0.1658, 0.2202]	<0.001	2587.002	0.1013 [0.0645, 0.1381]	<0.001	1583.625
SOFA Before Discharge				0.3343 [0.2873, 0.3813]	<0.001	1408.105

The logistic regression model, which included age and the SOFA score at ICU discharge, demonstrated that the SAPS II was the best predictor of ICU mortality based on its AIC values. Additionally, the SOFA score at ICU discharge provided a better fit for predicting mortality outside the ICU compared to other severity scoring systems such as SAPS II, SAPS III, and APACHE II.

To account for potential biases, SAPS II-adjusted mortality rates were calculated. For patients aged below 81 years, the adjusted ICU mortality rate was 13.5%, while for those aged 81 years and above, the rate was 19.8%, with a highly significant difference (p < 0.001).

Post-ICU mortality was also examined using the SOFA score at ICU discharge as the most reliable predictor. Adjusted post-ICU mortality rates were 8.0% for patients aged below 81 years and 9.3% for those aged 81 years and above, again showing a significant difference (p < 0.001).

## Discussion

Our data highlight the significant impact of age on the outcomes of critically ill patients, particularly within the ICU setting. Older adults, as represented by patients aged 81 years and above, exhibited worse survival outcomes compared to younger patients. Even after adjusting for severity scores, they still had significantly higher mortality rates both during ICU stays and after discharge. These findings underscore the importance of age as an independent risk factor for poor outcomes in critically ill patients.

The analysis demonstrated that the SAPS II score was the most reliable predictor of ICU mortality, while the final SOFA score provided a better assessment of mortality risk after ICU discharge. These results affirm the utility of established severity scoring systems in guiding clinical decision-making, particularly in high-risk groups like older adults.

Our study revealed that, for patients aged 81 years and above, mortality levels inside and outside of the ICU were comparable. Such persistently high mortality rates among older adults highlight their increased vulnerability, irrespective of the care setting. This finding raises an urgent need for special screening measures, tailored treatment plans, and post-discharge care for elderly individuals with acute illnesses and significant comorbidities.

By focusing on a substantial cohort of 692 patients aged 81 years and above, our study offers nuanced insights into mortality patterns and risk factors in this population. Prior studies, often limited to smaller samples, have suggested similar trends [14]. We were able to identify differences in mortality trends between ICU and post-ICU settings, emphasizing the compounded effect of age and illness severity on patient outcomes.

Our findings align with previous research emphasizing the complexity of care for older adults. For instance, Tsang et al. highlighted that many elderly ICU patients can survive hospital discharge and even six months post-discharge, with a significant number returning home without formal support [3]. While our study confirms that survival is possible, it also underscores the disproportionately higher adjusted mortality rates in older adults, particularly after discharge, emphasizing the need for robust discharge planning and follow-up care.

Other studies have similarly reported that older adults often present with higher illness severity at ICU admission, which correlates with increased mortality [15]. The elevated post-ICU mortality rates observed in our study are consistent with the findings that deficiencies in discharge planning, rehabilitation, or early interventions can contribute to preventable deaths [16]. Multidisciplinary approaches are critical in addressing modifiable risk factors during and after ICU care, potentially reducing post-discharge mortality.

The importance of pre-existing health conditions and illness severity at admission as mortality predictors has been extensively documented. Although Charlson comorbidity indices were available for only a small subset of patients in our study, our findings align with prior research emphasizing the role of the SOFA score as a reliable predictor of mortality [17]. This further highlights the need for comprehensive geriatric assessments in ICU settings to better identify and manage at-risk older adults.

Managing care for older adults in the ICU poses significant ethical and logistical challenges, especially in resource-limited settings [18]. Tailored approaches that prioritize both survival and quality of life are crucial [19]. For example, training programs aimed at equipping healthcare providers with skills in geriatric-focused care have been shown to improve outcomes for elderly ICU patients [20]. Such approaches include integrating geriatric-focused care strategies, emphasizing functional recovery, and introducing palliative care when appropriate. Previous studies [21,22] have shown that traditional ICU practices may not adequately address the needs of older adults, necessitating innovative models of care. For instance, integrating palliative care into ICU settings has been shown to reduce the burden of aggressive treatments that may not meaningfully improve outcomes [23]. The absence of detailed analysis of comorbidities, frailty, and social determinants of health further restricts our understanding of individual patient vulnerabilities and introduces potential biases regarding ICU practices and decisions [24].

This study has several limitations that warrant consideration. The retrospective, single-center design limits the generalizability of the findings, as variations in care practices, admission criteria, and resources across institutions were not accounted for. Additionally, the lack of longitudinal follow-up data precludes insights into long-term outcomes such as functional recovery, rehospitalization, or quality of life, which are particularly relevant for older adults. Moreover, the lack of data on DNR (do-not-resuscitate) orders could introduce bias, as these decisions often impact outcomes but were not evaluated in our study.

The overlap of our study period with the COVID-19 pandemic also introduces confounding factors. Changes in ICU protocols, resource allocation, and patient care practices during this time may have influenced observed mortality rates. Furthermore, the potential influence of technological and therapeutic advancements over the eight-year study period was not explicitly assessed. Finally, the inherent inclusion of age in severity scoring systems may have contributed to a self-reinforcing bias in the mortality predictions.

Future research should address these gaps by incorporating detailed comorbidity profiles, frailty assessments, and longitudinal data to explore long-term outcomes such as functional recovery and quality of life. Understanding the interplay between comorbidities, frailty, and social determinants of health could provide more nuanced insights into the vulnerabilities of older adults in critical care [25]. Additionally, the role of early physical therapy and rehabilitation in improving functional outcomes and reducing post-ICU mortality warrants further investigation [6].

The data emphasize the need for comprehensive risk assessment and management strategies tailored to critically ill older adults. Effective discharge planning, multidisciplinary interventions, and post-ICU monitoring are critical in mitigating modifiable risk factors. These approaches are particularly vital during public health crises like COVID-19, where older adults with pre-existing conditions face heightened risks [26].

## Conclusions

In conclusion, our study shows the significant impact of age and severity of illness on the outcomes of critically ill elderly patients within the ICU, even when adjusting to mortality risk. The findings emphasize the importance of developing geriatric-focused care strategies, including early palliative care integration and tailored interventions based on frailty and comorbidities to improve survival rates and quality of life. While our study has limitations, it has managed to highlight areas for future research, particularly regarding functional status and long-term outcomes post-ICU discharge which are essential for refining care strategies and ensuring better outcomes for this vulnerable population.

## References

[1] Rayman G, Akpan A, Cowie M, Evans R, Patel M, Posporelis S, Walsh K (2022). Managing patients with comorbidities: Future models of care. Future Healthc J.

[2] Guidet B, Vallet H, Boddaert J (2018). Caring for the critically ill patients over 80: A narrative review. Ann Intensive Care.

[3] Tsang J, Bloomfield K, Lawrey Y, Wu Z, Connolly MJ (2020). The very old in intensive care: Admission characteristics, mortality and supports needed at six months postdischarge. Australas J Ageing.

[4] Madotto F, McNicholas B, Rezoagli E, Pham T, Laffey JG, Bellani G (2021). Death in hospital following ICU discharge: Insights from the LUNG SAFE study. Crit Care.

[5] Inouye SK, Peduzzi PN, Robison JT, Hughes JS, Horwitz RI, Concato J (2025). Importance of functional measures in predicting mortality among older hospitalized patients. JAMA.

[6] Rydingsward JE, Horkan CM, Mogensen KM, Quraishi SA, Amrein K, Christopher KB (2016). Functional status in ICU survivors and out of hospital outcomes: A cohort study. Crit Care Med.

[7] Kodati R, Muthu V, Agarwal R (2022). Long-term survival and quality of life among survivors discharged from a respiratory ICU in North India: A prospective study. Indian J Crit Care Med.

[8] Hamsen U, Drotleff N, Lefering R, Gerstmeyer J, Schildhauer TA, Waydhas C (2020). Mortality in severely injured patients: Nearly one of five non-survivors have been already discharged alive from ICU. BMC Anesthesiol.

[9] Plotnikoff KM, Krewulak KD, Hernández L (2021). Patient discharge from intensive care: An updated scoping review to identify tools and practices to inform high-quality care. Crit Care.

[10] Vollam S, Dutton S, Lamb S, Petrinic T, Young JD, Watkinson P (2018). Out-of-hours discharge from intensive care, in-hospital mortality and intensive care readmission rates: A systematic review and meta-analysis. Intensive Care Med.

[11] Vollam S, Gustafson O, Young JD, Attwood B, Keating L, Watkinson P (2021). Problems in care and avoidability of death after discharge from intensive care: A multi-centre retrospective case record review study. Crit Care.

[12] Kelmenson DA, Held N, Allen RR (2017). Outcomes of ICU patients with a discharge diagnosis of critical illness polyneuromyopathy: A propensity-matched analysis. Crit Care Med.

[13] Gayat E, Cariou A, Deye N (2018). Determinants of long-term outcome in ICU survivors: Results from the FROG-ICU study. Crit Care.

[14] Vallet H, Schwarz GL, Flaatten H, De Lange DW, Guidet B, Dechartres A (2021). Mortality of older patients admitted to an ICU: A systematic review. Crit Care Med.

[15] Sakr Y, Jaschinski U, Wittebole X (2018). Sepsis in intensive care unit patients: Worldwide data from the intensive care over nations audit. Open Forum Infect Dis.

[16] Desai SV, Law TJ, Needham DM (2011). Long-term complications of critical care. Crit Care Med.

[17] Ferreira FL, Peres Bota D, Bross A, Mélot C, Vincent JL (2001). Serial evaluation of the SOFA score to predict outcome in critically ill patients. JAMA.

[18] Akdeniz M, Yardımcı B, Kavukcu E (2021). Ethical considerations at the end-of-life care. SAGE Open Med.

[19] Tringale M, Stephen G, Boylan AM, Heneghan C (2022). Integrating patient values and preferences in healthcare: A systematic review of qualitative evidence. BMJ Open.

[20] Aliberti MJ, Bailly S, Anstey M (2022). Tailoring treatments to older people in intensive care. A way forward. Intensive Care Med.

[21] Geen O, Rochwerg B, Wang XM (2021). Optimizing care for critically ill older adults. CMAJ.

[22] Jacobs JM, Rahamim A, Beil M (2024). Critical care beyond organ support: The importance of geriatric rehabilitation. Ann Intensive Care.

[23] Romano AM, Gade KE, Nielsen G (2017). Early palliative care reduces end-of-life intensive care unit (ICU) use but not ICU course in patients with advanced cancer. Oncologist.

[24] De Biasio JC, Mittel AM, Mueller AL, Ferrante LE, Kim DH, Shaefi S (2020). Frailty in critical care medicine: A review. Anesth Analg.

[25] Eggmann S, Luder G, Verra ML, Irincheeva I, Bastiaenen CH, Jakob SM (2020). Functional ability and quality of life in critical illness survivors with intensive care unit acquired weakness: A secondary analysis of a randomised controlled trial. PLoS One.

[26] Santos MM, Pereira IJ, Cuboia N (2023). Predictors of early and long-term mortality after ICU discharge in critically ill COVID-19 patients: A prospective cohort study. PLoS One.

